# A Preliminary Simulation Study of Dose-Guided Adaptive Radiotherapy Based on Halcyon MV Cone-Beam CT Images With Retrospective Data From a Phase II Clinical Trial

**DOI:** 10.3389/fonc.2020.574889

**Published:** 2020-09-29

**Authors:** Yuliang Huang, Haiyang Wang, Chenguang Li, Qiaoqiao Hu, Hongjia Liu, Jun Deng, Weibo Li, Ruoxi Wang, Hao Wu, Yibao Zhang

**Affiliations:** ^1^Key Laboratory of Carcinogenesis and Translational Research (Ministry of Education/Beijing), Department of Radiation Oncology, Peking University Cancer Hospital and Institute, Beijing, China; ^2^Department of Therapeutic Radiology, Yale University School of Medicine, New Haven, CT, United States; ^3^Helmholtz Zentrum München-German Research Center for Environmental Health (GmbH), Institute of Radiation Medicine, Neuherberg, Germany; ^4^Institute of Medical Technology, Peking University Health Science Center, Beijing, China

**Keywords:** Halcyon, megavoltage cone-beam CT, deformable image registration, adaptive radiotherapy, dose reconstruction

## Abstract

**Background and purpose:** To evaluate the feasibility of dose-guided adaptive radiotherapy (ART) based on deformable image registration (DIR) using fractional megavoltage cone-beam CT (MVCBCT) images from Halcyon system that uses identical beams for treatment and imaging and to retrospectively investigate the influence of anatomic changes on target coverage and organ-at-risk (OAR) sparing across various tumor sites.

**Materials and Methods:** Four hundred twenty-two MVCBCT images from 16 patients (three head and neck, seven thoracic, three abdominal, and three pelvic cases) treated in a phase II clinical trial for Halcyon were selected. DIR between the planning CT and daily MVCBCT image was implemented by Velocity software to create pseudo CT. To investigate the accuracy of dose calculation on pseudo CT, three evaluation patients with rescanned CT and adaptive plans were selected. Dose distribution of adaptive plans calculated on pseudo CT was compared with that calculated on the rescanned planning CT on the three evaluation patients. To investigate the impact of inter-fractional anatomic changes on target dose coverage and dose to OARs of the 16 patients, fractional dose was calculated and accumulated incrementally based on deformable registration between planning CT and daily MVCBCT images.

**Results:** Passing rates using 3 mm/3%/10% threshold local gamma analysis were 93.04, 96.00, and 91.68%, respectively, for the three evaluation patients between the reconstructed dose on pseudo CT (MVCBCT) and rescanned CT, where accumulated dose deviations of over 97% voxels were smaller than 0.5 Gy. Planning target volume (PTV) D95% and D90% (the minimum dose received by at least 95/90% of the volume) of the accumulated dose could be as low as 93.8 and 94.5% of the planned dose, respectively. OAR overdose of various degrees were observed in the 16 patients relative to the planned dose. In most cases, OARs' dose volume histogram (DVH) lines of accumulated and planned dose were very close to each other if not overlapping. Among cases with visible deviations, the differences were bilateral without apparent patterns specific to tumor sites or organs.

**Conclusion:** As a confidence building measure, this simulation study suggested the possibility of ART for Halcyon based on DIR between planning CT and MVCBCT. Preliminary clinical data suggested the benefit of patient-specific dose reconstruction and ART to avoid unacceptable target underdosage and OAR overdosage.

## Introduction

Anatomic changes during the treatment course are one of the leading contributors of delivered dose uncertainties in radiotherapy, potentially causing underdosage to the targets or overdosage to organs at risk (OAR) relative to the planned dose distribution (1, 2). Adaptive radiotherapy (ART) based on periodical replanning has been reported as beneficial for patients with various diseases (3–5). However, as ART is a labor-intensive and time-consuming process, it is clinically desirable to strike a balance between implementation efficiency and treatment outcomes by deciding an optimal replanning time point, which is preferably dose-guided, rather than depending on conventional subjective experience (6) or anatomic observation (7).

Daily dose reconstruction and accumulation based on deformable image registration (DIR) between planning CT and pretreatment images are one of the major ART approaches (8–10). Although DIR-based ART has been tested on kV CT (11, 12), megavoltage (MV) CT (13, 14), and kV cone-beam CT (CBCT) (15–17), the imaging frequency using those modalities was sometimes sacrificed to reduce concomitant extra dose and secondary cancer risk on the conventional image-guided radiotherapy (IGRT) platforms (18), providing incomplete records for dose reconstruction and analysis. The MV cone-beam CT (MVCBCT) on Halcyon system (Varian Medical Systems, Pala Alto, CA) uses identical 6-MV flattening-filter-free (FFF) treatment beams for imaging, which enables accurate computation, collective optimization, and automated incorporation of the imaging and treatment doses on Varian Eclipse treatment planning system (V.15.1 or later), instead of complex manual simulations and validations on other platforms (19). In addition, fractional image guidance has been made compulsory on Halcyon, which also takes a shorter scanning time (~15 s per MVCBCT acquisition) than the conventional CBCT due to a faster gantry rotation speed (up to 4 RPM). These new features are quite different from those of the MVision system (Siemens Medical Solutions, Concord, CA) (20), potentially making Halcyon a suitable system for ART. A previous phantom-based study has evaluated the accuracy of DIR utilizing Halcyon MVCBCT images and reported that the accuracy was acceptable under specific conditions (21). However, clinical data-based study on ART utilizing Halcyon MVCBCT has not yet been reported.

This work aims to test the feasibility of fractional dose reconstruction and accumulation on the Halcyon IGRT system based on MVCBCT, potentially making dose-guided ART possible on this new platform. The reconstructed dose analysis involved 422 MVCBCT of 16 patients who were treated in the phase II clinical trial for Halcyon's clearance by Chinese FDA (CFDA). These pioneer clinical data and preliminary experience on Halcyon application in China may assist more informed decisions of ART using image modalities such as MVCBCT.

## Materials and Methods

### Patient Data

The data of 16 patients recruited in an ethically approved Halcyon phase II clinical trial (IRB#2017QX03) with signed informed consent were retrospectively included in this study. All these patients received fractional pretreatment MVCBCT imaging on Halcyon, including three head and neck, seven thoracic, three abdominal, and three pelvic cases. In addition to fractional MVCBCT images, the physician-approved planning CT images with contours, clinical treatment plans and records, ART rescanned CT images, and replans (if any) were also used in this study. Dose distributions were calculated on Eclipse V15.6 using AAA algorithm.

### Deformable Image Registration

For each patient, the initial planning CT was registered to the fractional Halcyon MVCBCT images using DIR algorithm implemented on Varian Velocity 4.0 software, which has demonstrated satisfactory accuracy on other imaging modalities (22). The DIR-generated pseudo CT combined information of both Hounsfield unit (HU) values from the planning CT and the daily anatomy from the MVCBCT images. Since the maximum reconstructed field of view of Halcyon MVCBCT was 27.6 cm × 27.6 cm × 28 cm, which might be smaller than the patient size, the MVCBCT was patched with the outside of the patient from the planning CT. Taking into account the image-guided couch shift, a rigid pre-alignment was conducted before DIR. The DIR procedure adopted free-form B-spline algorithm with 5 mm × 5 mm × 5 mm grid spacing and mutual information as the objective function. Manual review and necessary modification were performed for each patient. The inverse DIR transformation was used for dose mapping back to planning CT. The whole process took 30 min on average for one patient per fraction.

### Evaluation of Megavoltage Cone-Beam CT-Based Dose Reconstruction

Following Held's method (23), an electron density phantom (Sun Nuclear, GammexTM Technology) with plugins of known electron density relative to water (RED), was scanned by Halcyon MVCBCT system to obtain the HU-to-RED curve, which was then applied to calibrate the MVCBCT image of the Catphan604 phantom (The Phantom Laboratory, Salem, NY) for dose calculation evaluation. The dose distributions of a 10 cm × 10 cm 6-MV flattening-filter-free open field were calculated on the planning CT and MVCBCT images, respectively, for comparison.

### Evaluation of Deformable Image Registration Accuracy

To investigate the accuracy of dose calculation on pseudo CT, three evaluation patients with ART history were selected, making use of the anatomic similarities between the rescanned CT and the MVCBCT of the same day. These historical adaptive imaging and treatments were decided by physicians based on their clinical experience and anatomical observation. As reference, the dose distributions of the adaptive plans were calculated on the rescanned CTs. As comparison, the dose distributions of the adaptive plans were also calculated on the pseudo CT, created by registering the initial planning CT to the MVCBCT taken on the same day of the rescanned CTs. To avoid the interference from patient setup errors, the pseudo CT was rigidly registered to the rescanned CT before dose calculation. As evaluations, gamma analysis was performed using an in-house code under criteria of 3 mm/3/10% threshold local dose. Histograms of both absolute and relative dose differences were computed for displaying.

### Reconstructed Dose Accumulation on Halcyon

To investigate the impact of inter-fractional anatomic changes on target dose coverage and dose to OARs of the 16 patients who were treated in the phase II clinical trial of Halcyon, fractional dose was calculated and accumulated incrementally based on deformable registration between planning CT and daily MVCBCT images. Specifically, after the treatment plan was calculated on the pseudo CT of each fraction, the dose distribution was mapped back to the planning CT for accumulation, using the inverse deformation vector field. For the three evaluation patients with ART histories, the corresponding initial and the adaptive treatment plans were chosen to calculate the fractional dose before and after replanning, respectively. D95% and D90% (the minimum dose received by at least 95/90% of the volume) of the planning target volume (PTV) as well as dose to OARs at various anatomic sites were calculated and compared between the planned and accumulated doses.

## Results

### Dose Reconstruction Accuracy

**Figure 1:**
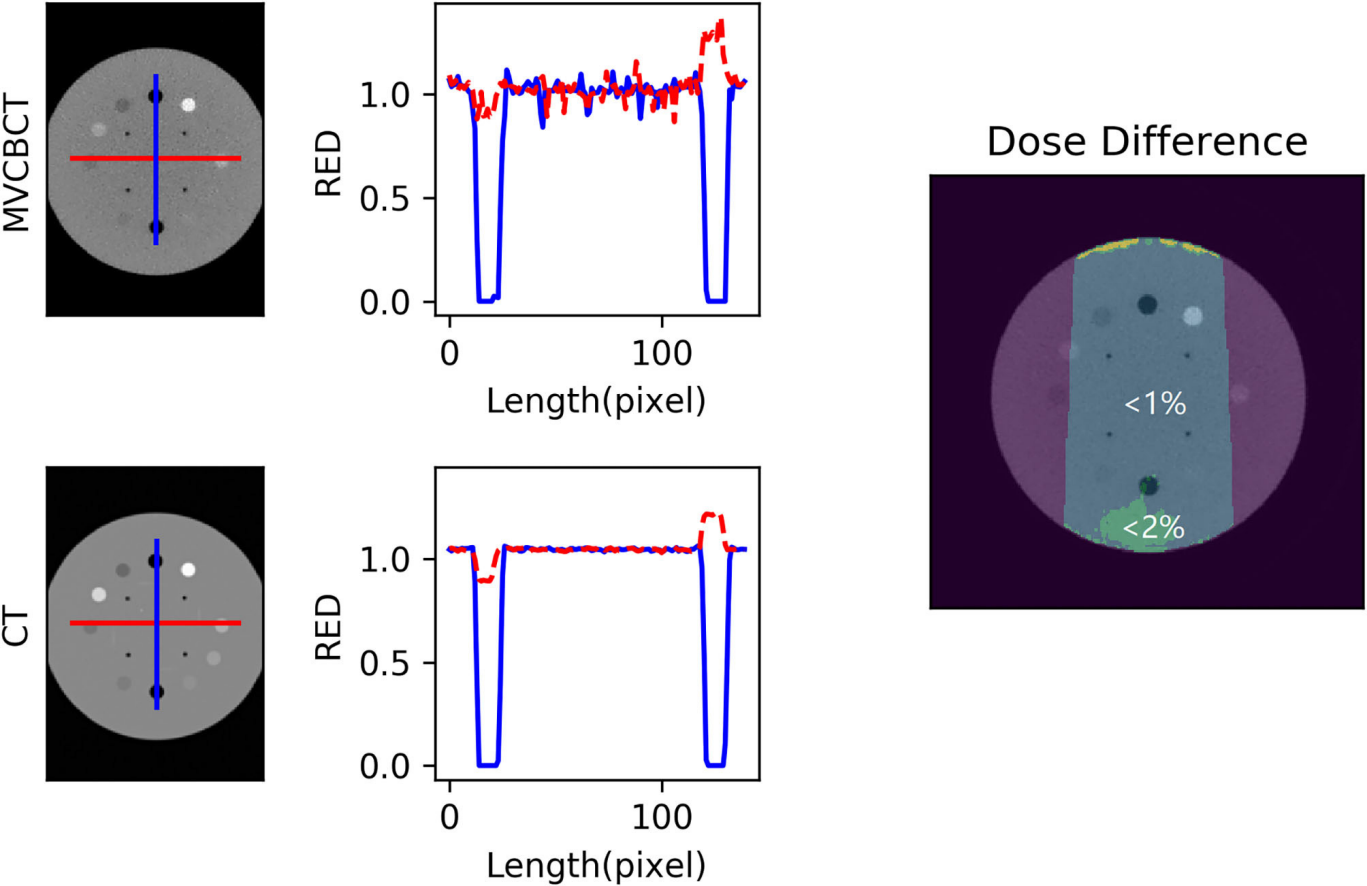
Image comparison of planning CT and calibrated megavoltage cone-beam CT (MVCBCT) of a Catphan604 phantom. The electron density relative to water (RED) profile along central vertical (blue) and horizontal (red) lines on one axial slice was plotted. Regions with <1 and <2% dose difference with respect to the planned dose were shown in the right column subfigure. The mean dose difference in the top surface region was 5.43%.

Figure 1 compares the reconstructed RED and dose distributions on planning CT and calibrated MVCBCT images of the Catphan604 phantom, respectively. The blue and red lines in the left and middle columns show the RED profiles along the central vertical and horizontal lines of the same axial slice of the planning CT and MVCBCT images, respectively. The right column graph shows that the dose differences in most regions were within 1 and 2% with respect to the planned dose. The maximum dose difference was observed in a small area on the top surface, where the regional mean difference was 5.43%.

### Deformable Image Registration Accuracy

**Figure 2:**
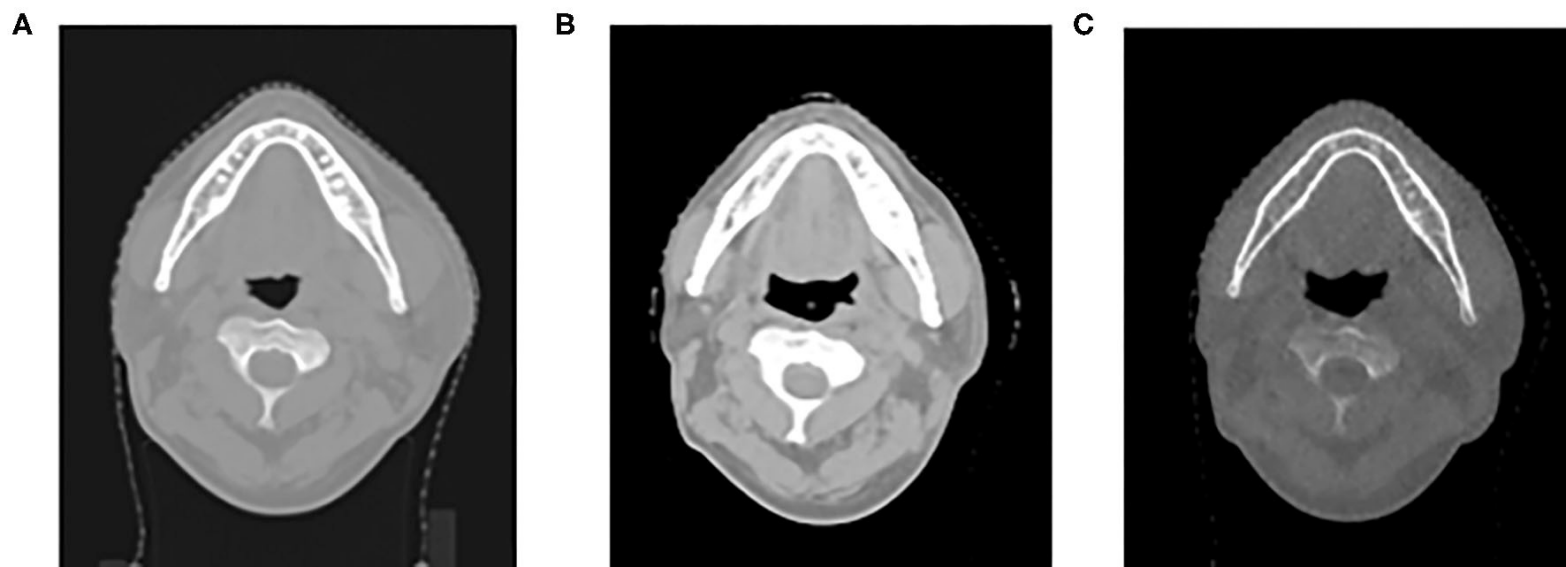
Planning CT **(A)**, rescanned CT **(B)**, and corresponding megavoltage cone-beam CT (MVCBCT) image on the same day of rescanned CT **(C)** of one example patient.

**Figure 3:**
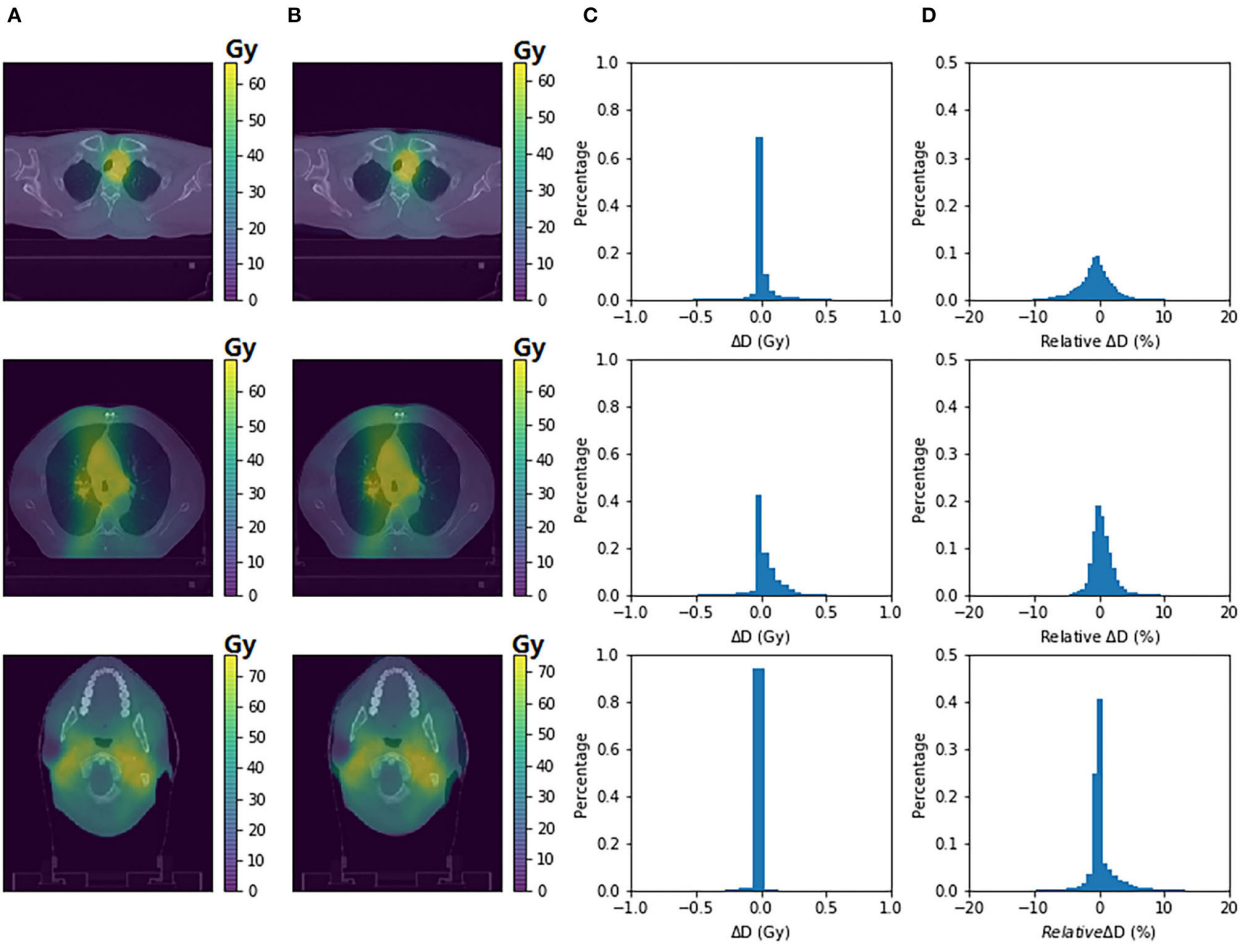
Dosimetric accuracy of deformable image registration (DIR)-based dose reconstruction. The dose distribution of adaptive plans as calculated on rescanned CT **(A)** and pseudo CT **(B)** was demonstrated on axial planes for three patients with replanning history, respectively. Histograms of absolute/relative dose differences between the two dose distributions were shown on columns **(C,D)**, respectively.

Figure 2 demonstrates the planning CT (Figure 2A), rescanned CT (Figure 2B), and corresponding MVCBCT images on the same day of rescanned CT (Figure 2C) of one example patient. Anatomic similarities can be observed between Figures 2B,C, which were both different from Figure 2A before radiotherapy started. Dose distribution of adaptive plans were computed on the rescanned CT and the pseudo CT obtained by deforming the planning CT to the daily MVCBT image, respectively. Figure 3 compares the dose distribution of three evaluation patients with adaptive plans as calculated on the rescanned CT [reference, column (a)] and the corresponding deformed pseudo CT [recalculated, column (b)] of the same day, respectively. Histograms of absolute/relative dose differences between the reference and recalculated doses were shown on columns (c) and (d), respectively. The relative dose differences were calculated against the local dose with 10% threshold, in accordance with the gamma criteria. Passing rates of 3 mm/3/10% threshold local gamma analysis were 93.04, 96.00, and 91.68% for the three patients from the top down, respectively.

### Target Dose Coverage

Taking the corresponding planned dose as reference, the normalized PTV D95% of the cumulated dose for the 16 patients was 1.001, 0.977, 0.998 (head and neck), 0.973, 0.992, 0.997, 0.994, 0.964, 0.999, 0.974 (thorax), 0.958, 0.984, 0.938 (abdomen), and 0.983, 0.983, 0.987 (pelvis), respectively. The normalized D90% of the cumulated dose was 1.001, 0.987, 1.000 (head and neck), 0.987, 0.998, 0.998, 1.001, 0.979, 1.001, 0.989 (thorax), 0.976, 0.993, 0.945 (abdomen), and 0.993, 0.985, 0.993 (pelvis), respectively.

### Organ-at-Risk Sparing

**Figure 4:**
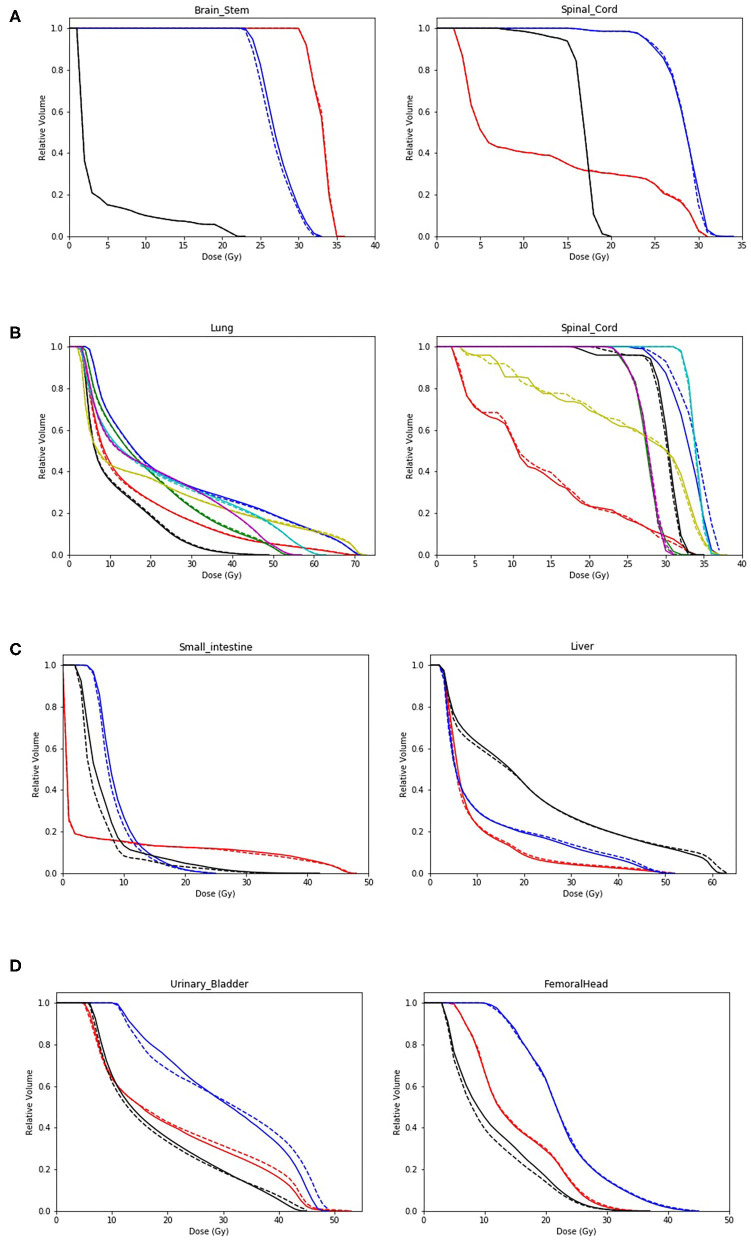
Dose volume histogram (DVH) comparison between the planned and reconstructed doses of two organs at risk (OARs) per anatomic site. Head and neck: brain stem and spinal cord **(A)**; Thorax: lung and spinal cord **(B)**; Abdomen: small intestine and liver **(C)**; and Pelvis: urinary bladder and femoral head **(D)**. Solid and dotted lines of the same color indicate the cumulated and planned dose, respectively, for a specific patient.

The dose volume histograms (DVHs) of two representative OARs within the MVCBCT imaging field were selectively plotted for each group as examples. Figures 4A–D display the DVHs of the brain stem and spinal cord for the three head and neck patients, lung and spinal cord for the seven thoracic patients, small intestine and liver for the three abdominal patients, and urinary bladder and femoral head for the three pelvic patients, respectively. Solid and dotted lines of the same color indicate the cumulated and planned dose, respectively, for a specific patient.

## Discussion

Using similar methods of dose validation applied on Siemens system (23), the phantom-based evaluation using Halcyon MVCBCT displayed comparable dosimetric validation results (Figure 1). Essential for the DIR-based ART, registration accuracy was preliminarily evaluated as potentially feasible specifically for the Halcyon MVCBCT system based on the anatomic similarities between rescanned planning CT and the MVCBCT acquired on the same day (21). Previous study (21) evaluated the geometric accuracy of DIR on Halcyon MVCBCT using virtual phantom simulations and a non-commercial software. This work further demonstrated the dosimetric accuracy of DIR-based dose reconstruction based on phantom and real patient data, providing more clinical relevance. By comparing the dose distributions of adaptive plans on rescanned CT (gold standard) and on the pseudo CT created by registering initial CT to the MVCBCT images of the same day, it was observed that the gamma passing rates for the three patients were all above 91.68%, comparable to the range of 94.3 ± 5.4% as reported for DIR between CT and kV CBCT (15). Moreover, as shown in Figure 3, an overwhelmingly large proportion (>97 or 90%) of pixels displayed absolute dose differences smaller than 0.5 Gy or 3% of the reference dose respectively. Based on the phantom validation and preliminary clinical data, the accuracy of dose reconstruction on the Halcyon MVCBCT images using Velocity program is considered as potentially acceptable and was consequently used to analyze the 16 patients in this study.

One major concern of inter-fractional anatomic variation is the associated uncertainties of delivered dose to the targets, which can be effectively monitored by dose reconstruction on Halcyon MVCBCT. It was noticed that most PTV D95% and D90% tended to be slightly lower than the planned values. Considering the significant correlation of D95% and D90% with locoregional tumor recurrence (24), DIR-based dose monitoring using fractional Halcyon MVCBCT images can be beneficial for tumor control.

Managing dose deviations to OARs induced by inter-fractional anatomic changes was also possible using Halcyon MVCBCT images. Due to the various tumor sites involved in this study, only two representative OARs were selectively displayed per tumor site in Figure 4. In most cases, DVH lines of cumulated and planned dose were very close to each other if not overlapping. Among cases with visible deviations, the differences were bilateral without apparent patterns specific to tumor sites or organs. The only exception was the consistently increased dose to small intestine in the low-dose region for all abdominal cases with respect to planned dose, as shown in Figure 4C. The unpredictable randomness of dose deviations indicated that dose-guided ART might be more reliable than general experience or anatomic observation.

Dosimetric deviations observed in both targets and OARs demonstrated the effectiveness of dose reconstruction based on Halcyon MVCBCT using a commercially available DIR system, underscoring the necessity of ART for some patients. The feasibility of dose-guided ART has been tested by simulation of preliminary clinical data across different tumor sites on the new Halcyon platform. The compatibility between Eclipse and Velocity systems from the same vendor may further facilitate the potential clinical application of ART on Halcyon platform.

As a retrospective study, this work is limited by the small number of patients, as well as relatively lower image quality of MVCBCT than kV images. Although a recent study claimed sufficient soft-tissue contrast of Halcyon MVCBCT (25), the accuracy could be further improved by using the iterative kV CBCT (iCBCT) available on Halcyon V.2.0, which is worthy of further studies in the future.

## Conclusion

As a confidence building measure, this simulation study suggested the possibility of ART for Halcyon based on DIR between planning CT and MVCBCT using a commercially available software.

## Data Availability Statement

The datasets presented in this article are not readily available because Policy of Peking University Cancer Hospital. Requests to access the datasets should be directed to YZ, zhangyibao@pku.edu.cn.

## Ethics Statement

The studies involving human participants were reviewed and approved by Ethics Committee of Beijing Cancer Hospital. The patients/participants provided their written informed consent to participate in this study.

## Author Contributions

YH and HWa contributed equally to this work and were responsible for data acquisition and analyses. CL, QH, and HL contributed to performing deformable image registration. JD, WL, and RW were responsible for reviewing data analysis result. HWu and YZ were responsible for designing methodology and reviewing the whole research. All authors contributed to the article and approved the submitted version.

## Conflict of Interest

The authors declare that this study received funding from Varian Medical Systems. The authors declare that the research was conducted in the absence of any commercial or financial relationships that could be construed as a potential conflict of interest.
